# Supplementary material for: When One’s Not Enough: Colony Pool-Seq Outperforms Individual-Based Methods for Assessing Introgression in Apis mellifera mellifera
Source: Insects. 2023 Apr 27;14(5):421. doi: 10.3390/insects14050421 (PMC10231099; doi:10.3390/insects14050421)
Supplement: Supplementary file 1 [file insects-14-00421-s001.zip › Supplementary_files_information_when_ones_not_enough-proofread.pdf]

Supplementary Materials

When One's Not Enough: Colony Pool-Seq Outperforms Individual-Based Methods for Assessing Introgression in *Apis mellifera mellifera*

Victoria G. Buswell [1,2,*], Jonathan S. Ellis [1], J. Vanessa Huml [1], David Wragg [3,4], Mark W. Barnett [4], Andrew Brown [5], The Scottish Beekeepers Association Citizen Science Group and Mairi E. Knight [1]

1 School of Biological and Marine Sciences, University of Plymouth, Drake Circus, Plymouth PL4 8AA, UK

2 Information and Computational Sciences, The James Hutton Institute, Dundee DD2 5DA, UK

3 The Roslin Institute and Royal (Dick) School of Veterinary Studies, University of Edinburgh, Easter Bush Campus, Roslin EH25 9RG, UK

4 Beebytes Analytics CIC, Roslin Innovation Centre, Easter Bush Campus, Roslin EH25 9RG, UK

5 B4, Newton Farm Metherell, Cornwall, Callington PL17 8DQ, UK

* Correspondence: victoria.buswell@hutton.ac.uk

S1. GATK best practices pipeline and vcftools iterative filtering

Haplotype Caller was used to create the genomic variant call format (gvcf) files for every sample (both colony and individual) [1]. Then GATK's GenomicsDBImport was used to create two intermediate databases, one for colony samples and one for individual samples. This was followed by the creation of a vcf per chromosome, one for colony samples and one for individual samples, using GATK's GenotypeGVCFs. The per chromosome vcfs were merged into one vcf containing all samples and all sites using GatherVcfs [1]. Using GATK's SelectVariants command, indels were removed and only SNP variants were kept.

After GATK best practices a robust filtering method for ddRADseq data designed by O'Leary et al., (2018)[2] was performed which mitigates errors in downstream data analysis that can be caused by allelic dropout. (Table S12)

Table S12. The GATK hard filtering thresholds and the O'Leary et al (2018) RADseq filtering pipeline.

**Filter name and programme**	**Filter description**	**Threshold**
QualByDepth (GATK)	Variant confidence divided by the unfiltered depth. Filtered at GATK recommended value.	< 2
Quality (GATK)	Variant quality confidence. Filtered at GATK recommended value.	< 30
FisherStrand (GATK)	Phred-scaled probability of strand bias. Filtered at GATK recommended value.	> 60

StrandOddsRatio (GATK)	Strand bias test that compensates for where FisherStrand filter penalises variants at the end of exons. Filtered at GATK recommended value.	> 3
RMSMAppingQuality (GATK)	Root mean square mapping quality over all reads at each site. Filtered at GATK recommended value.	< 40
MappingQualityRankSumTest (GATK)	Rank sum test for mapping qualities. This compares mapping qualities of the reads supporting the reference allele at the alternate allele. Filtered at GATK recommended value.	< - 12.5
ReadPosRankSumTest (GATK)	The rank sum test for site position within reads. This compares whether positions of the reference and alternate alleles are different within the reads. Filtered at GATK recommended value.	< - 8.0
--remove-indels (vcftools)	Remove any insertions or deletions in the data, leaving only SNPs	All removed
--maxDP and –minDP (vcftools)	Filters sites based on read depth, removing any below the minimum threshold and above the maximum threshold.	>5 and <500
--minQ (vcftools)	Retain sites with quality value above this threshold.	>20
--max-missing (vcftools)	SNPs excluded based on a proportion of missingness across all samples	>0.5
--missing-indv (vcftools)	Samples excludes based on a proportion of missing SNPs	<0.9
--max-missing (vcftools)	SNPs excluded based on a proportion of missingness across all samples	>0.6
--missing-indv (vcftools)	Samples excludes based on a proportion of missing SNPs	<0.7
--max-missing (vcftools)	SNPs excluded based on a proportion of missingness across all samples	>0.7
Data filtering continued		
--missing-indv (vcftools)	Samples excludes based on a proportion of missing SNPs	<0.5
--missing-indv (vcftools)	Samples excludes based on a proportion of missing SNPs	>0.25
--max-missing (vcftools)	SNPs excluded based on a proportion of missingness across all samples	<0.95
Filter_monomorphic.py	Custom python code that removed any monomorphic sites	All removed

S2. ABBA BABA calculations and information

The ABBA BABA approach was developed in a series of papers [3–6]. ABBA and BABA patterns are calculated using allele frequencies at fixed sites in the outgroup [5,7]:

$$ABBA = (1 - P1) \times P2 \times P3 \times 1 - Po$$

$$BABA = P1 \times (1 - P2) \times P3 \times 1 - Po$$

Paterson's D is calculated using the sum of ABBA and BABA patterns across all SNPs:

$$D = \frac{\sum(ABBA) - \sum(BABA)}{\sum(ABBA) + \sum(BABA)}$$

When D deviates from zero it can be indicative of introgression between populations. An excess of ABBA patterns (introgression between P2 and P3) and would result in a D value > 1 and an excess of BABA sites (introgression between P1 and P3) will result in a D value <1. In this study significant positive D values would indicate introgression between, putative *A. m. mellifera* honey bees (P2) from the South West of England and C lineage honey bees (P3) (Figure 2). To test whether the D statistics significantly varies from zero, Z-scores and P-values were calculated (results in supplementary tables S2 and S3) [3–6].

The related *f* statistic used to estimate over all proportion of admixture was calculated:

$$f = \frac{\sum(ABBA) - \sum(BABA)}{\sum(ABBA) + \sum(BABA)}$$

Where:

$$ABBA_{numerator} = (1 - P1) \times P2 \times P3a$$

$$BABA_{numerator} = P1 \times (1 - P2) \times P3a$$

$$ABBA_{Denominator} = (1 - P1) \times P3b \times P3a$$

$$BABA_{Denominator} = P1 \times (1 - P3b) \times P3a$$

To calculate the D statistic and *f* statistic in the pooled data, colony level allele frequencies were calculated at each SNP using the AD and DP fields from the info column in the vcf file, this is the same method employed for pooled data by poolfstat [8]. The individual worker RADseq calculations were performed in the software package Dsuite [9]. For the individual analysis Dsuite performs

another related *f* statistic, the *f* 4-ratio [6]. The *f* 4-ratio, just like *f*, estimates the proportion of admixture between P2 and P3 but here the result is a ratio where represents the admixture from P2 to P3 and 1-α the admixture from P3 to P2 (Figure S1)

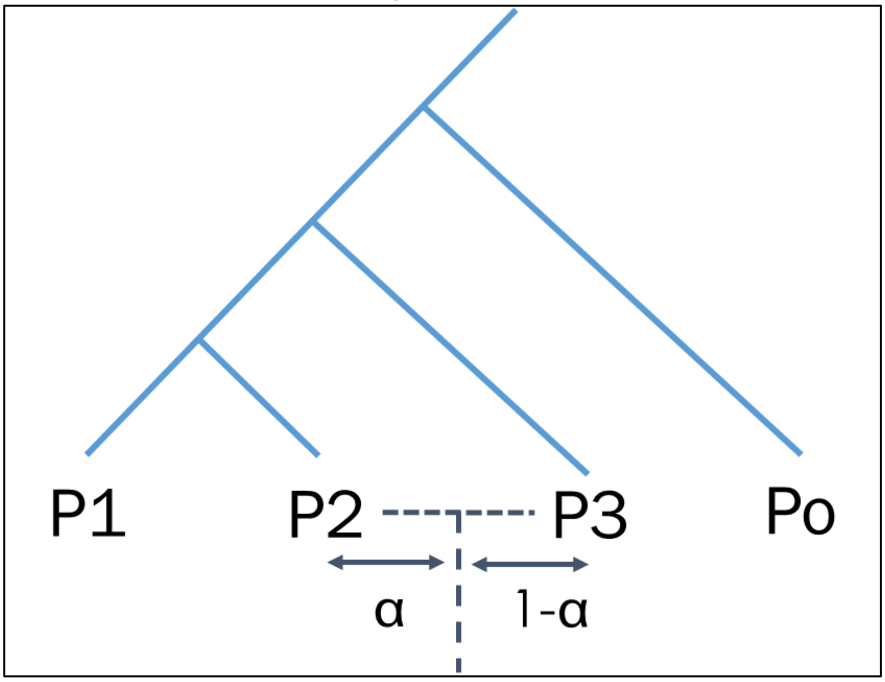

Figure S1. Overview of South West England ddRADseq samples and subspecies standards bioinformatics workflows. Samples were processed based on the sampling approach

The admixture ratio (α) between P2 and P3 is calculated by again splitting P3 into two groups, P3a and P3b and replacing P2 with P3b to compare observed ABBA BABA patterns to patterns of complete admixture. Specifically, if P3 were to be split into P3a and P3b, P3a and P3b represent the same subspecies and the admixture would be a proportion of 1.0, total admixture. Dsuite [9] calculates the f4 ratio as:

$$f4ratio = \frac{\sum(P3a - 1) \times \sum(P2 - P1)}{\sum(P3a - 1) \times \sum(P3b - P1)}$$

Dsuite splits P3 by randomly sampling alleles from P3 at each SNP. D suite also calculates normalised Z scores and P-values.

To test for significance Z-scores are generated using block jack-knifing, which accounts for the non-independence of linked sites. During block jack-knifing data are divided into blocks of a particular genomic distance or number of SNPs, and the D-statistic is calculated for each of these blocks. Then,

the overall D is compared to the standard error of D resulting from the blocks, and a Z-score is calculated. Importantly, RADseq data can contain linked groups of SNPs and this can confound the standard error of jackknife block. Additionally, implementing Z-scores assumes that the data is normally distributed, but often, D statistics resulting from jackknife blocks may not be. To obtain an approximately normally distributed standard error the variation of D over the blocks is calculated, multiplied by the number of blocks and the square root of that number is taken [3–5]. From this a Z-score is calculated:

$$Z\ score = \frac{D}{normally\ distributed\ \ Standard\ Error\ of\ D}$$

and then a p-value to estimate significance:

$$p\ value = 2 \times Log\ of\ the\ cumlative\ distibution\ function\ (-Z\ score)$$

S3. Sense check of SNP array results

To sense check the SNP array results a comparison of the ADMIXTURE Q values for the subspecies standards were compared back to the results from Henriques *et al* (2018). These subspecies standard data were generated by Pinto *et al* (2014) and examined in Munoz *et al.*, (2015) and Henriques *et al.*, (2018)[10–12].

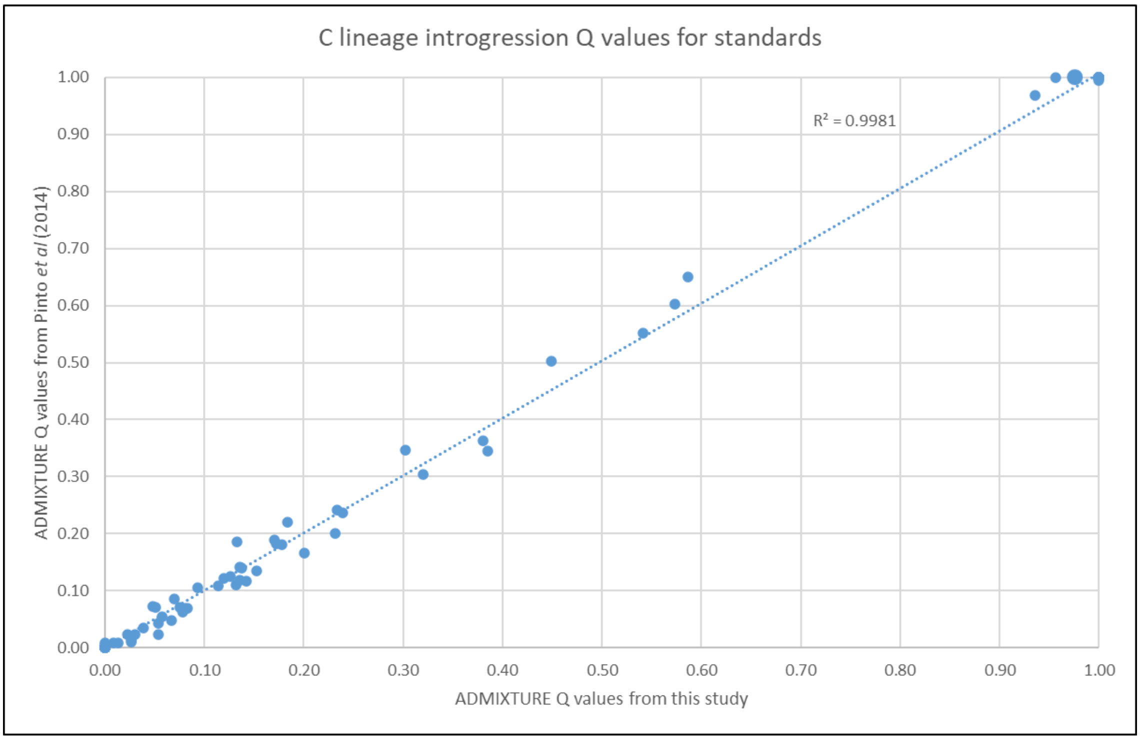

Figure S2. Comparison of results for subspecies standards that accompany the SNP array. ADMIXTURE Q value results from this experiment plotted against the results from the Henriques *et al* (2018).

References

(1) Poplin, R.; Ruano-Rubio, V.; DePristo, M. A.; Fennell, T. J.; Carneiro, M. O.; Van der Auwera, G. A.; Kling, D. E.; Gauthier, L. D.; Levy-Moonshine, A.; Roazen, D. Scaling Accurate Genetic Variant Discovery to Tens of Thousands of Samples. *BioRxiv* **2017**, 201178.

(2) O'Leary, S. J.; Puritz, J. B.; Willis, S. C.; Hollenbeck, C. M.; Portnoy, D. S. These Aren't the Loci You're Looking for: Principles of Effective SNP Filtering for Molecular Ecologists. *Mol. Ecol.* **2018**, *27* (16), 3193–3206.

(3) Green, R. E.; Krause, J.; Briggs, A. W.; Maricic, T.; Stenzel, U.; Kircher, M.; Patterson, N.; Li, H.; Zhai, W.; Fritz, M. H.-Y. A Draft Sequence of the Neandertal Genome. *Science (80-.).* **2010**, *328* (5979), 710–722.

(4) Reich, D.; Green, R. E.; Kircher, M.; Krause, J.; Patterson, N.; Durand, E. Y.; Viola, B.; Briggs, A. W.; Stenzel, U.; Johnson, P. L. F. Genetic History of an Archaic Hominin Group from Denisova Cave in Siberia. *Nature* **2010**, *468* (7327), 1053–1060.

(5) Durand, E. Y.; Patterson, N.; Reich, D.; Slatkin, M. Testing for Ancient Admixture between Closely Related Populations. *Mol. Biol. Evol.* **2011**, *28* (8), 2239–2252.

(6) Patterson, N.; Moorjani, P.; Luo, Y.; Mallick, S.; Rohland, N.; Zhan, Y.; Genschoreck, T.; Webster, T.; Reich, D. Ancient Admixture in Human History. *Genetics* **2012**, *192* (3), 1065–1093.

(7) Martin, S. H.; Davey, J. W.; Jiggins, C. D. Evaluating the Use of ABBA–BABA Statistics to Locate Introgressed Loci. *Mol. Biol. Evol.* **2015**, *32* (1), 244–257.

(8) Gautier, M.; Vitalis, R.; Flori, L.; Estoup, A. F-Statistics Estimation and Admixture Graph Construction with Pool-Seq or Allele Count Data Using the R Package Poolfstat. *Mol. Ecol. Resour.* **2022**, *22* (4), 1394–1416.

(9) Malinsky, M.; Matschiner, M.; Svardal, H. Dsuite - Fast D -statistics and Related Admixture Evidence from VCF Files. *Mol. Ecol. Resour.* **2021**, *21* (2), 584–595.

(10) Henriques, D.; Parejo, M.; Vignal, A.; Wragg, D.; Wallberg, A.; Webster, M. T.; Pinto, M. A. Developing Reduced SNP Assays from Whole-genome Sequence Data to Estimate Introgression in an Organism with Complex Genetic Patterns, the Iberian Honeybee (*Apis Mellifera Iberiensis*). *Evol. Appl.* **2018**, *11* (8), 1270–1282.

(11) Pinto, M. A.; Henriques, D.; Chávez-Galarza, J.; Kryger, P.; Garnery, L.; van der Zee, R.; Dahle, B.; Soland-Reckeweg, G.; de la Rúa, P.; Dall' Olio, R.; Carreck, N. L.; Johnston, J. S. Genetic Integrity of the Dark European Honey Bee (*Apis Mellifera Mellifera*) from Protected Populations: A Genome-Wide Assessment Using SNPs and MtDNA Sequence Data. *J. Apic. Res.* **2014**, *53* (2), 269–278.

(12) Muñoz, I.; Henriques, D.; Jara, L.; Johnston, J. S.; Chávez-Galarza, J.; De La Rúa, P.; Pinto, M. A. SNPs Selected by Information Content Outperform Randomly Selected

Microsatellite Loci for Delineating Genetic Identification and Introgression in the Endangered Dark European Honeybee (*Apis Mellifera Mellifera*). *Mol. Ecol. Resour.* **2017**, *17* (4), 783–795.
